# The emotional face of anorexia nervosa: The neural correlates of emotional processing

**DOI:** 10.1002/hbm.25417

**Published:** 2021-03-19

**Authors:** Daniel Halls, Monica Leslie, Jenni Leppanen, Felicity Sedgewick, Simon Surguladze, Leon Fonville, Katie Lang, Mima Simic, Dasha Nicholls, Steven Williams, Kate Tchanturia

**Affiliations:** ^1^ Institute of Psychiatry, Psychology and Neuroscience (IoPPN), Psychological Medicine, King's College London (KCL) London UK; ^2^ Centre for Contextual Behavioural Science University of Chester Chester UK; ^3^ Centre for Neuroimaging Sciences King's College London London UK; ^4^ School of Education University of Bristol Bristol UK; ^5^ Division of Brain Sciences Imperial College London London UK; ^6^ King's College London (KCL) Institute of Psychiatry, Psychology and Neuroscience (IoPPN), Department of Psychology London UK; ^7^ South London and Maudsley NHS Foundation Trust London UK; ^8^ Psychology Department Illia State University Tbilisi Georgia

**Keywords:** anorexia nervosa, emotional faces, emotional processing, functional magnetic resonance imagining, psychophysical interaction

## Abstract

Social–emotional processing difficulties have been reported in Anorexia Nervosa (AN), yet the neural correlates remain unclear. Previous neuroimaging work is sparse and has not used functional connectivity paradigms to more fully explore the neural correlates of emotional difficulties. Fifty‐seven acutely unwell AN (AAN) women, 60 weight‐recovered AN (WR) women and 69 healthy control (HC) women categorised the gender of a series of emotional faces while undergoing Functional Magnetic Resonance Imaging. The mean age of the AAN group was 19.40 (2.83), WR 18.37 (3.59) and HC 19.37 (3.36). A whole brain and psychophysical interaction connectivity approach was used. Parameter estimates from significant clusters were extracted and correlated with clinical symptoms. Whilst no group level differences in whole brain activation were demonstrated, significant group level functional connectivity differences emerged. WR participants showed increased connectivity between the bilateral occipital face area and the cingulate, precentral gyri, superior, middle, medial and inferior frontal gyri compared to AAN and HC when viewing happy valenced faces. Eating disorder symptoms and parameter estimates were positively correlated. Our findings characterise the neural basis of social–emotional processing in a large sample of individuals with AN.

## Graphical abstract

Social–emotional processing difficulties have been reported in Anorexia Nervosa (AN), yet the neural correlates remain unclear. Using functional imaging and a functional connectivity approach, 57 acutely unwell AN (AAN) women, 60 weight‐recovered AN (WR) women and 69 healthy control (HC) women categorised the gender of a series of emotional faces. WR participants showed increased connectivity between the bilateral occipital face area and the cingulate, precentral gyri, superior, middle, medial and inferior frontal gyri compared to AAN and HC when viewing happy valenced faces which was correlated to eating disorder symptoms.

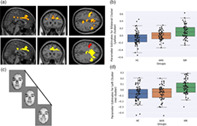

## 1 INTRODUCTION

Anorexia Nervosa (AN) is a life‐threatening eating disorder, characterised by low body mass index (BMI) and intense fear of weight gain, with undue influence placed on body shape and weight (APA, 2013). AN has a 1–4% incidence rate amongst European women (Keski‐Rahkonen & Mustelin, 2016) and is associated with a significantly elevated mortality rate (Arcelus, Mitchell, Wales, & Nielsen, 2011). Despite AN having a significant burden on health outcomes (Ágh et al., 2016), its neurobiology is poorly understood, especially when compared to other severe psychiatric disorders (Berner et al., 2019; Kaye, Wierenga, Bailer, Simmons, & Bischoff‐Grethe, 2013).

Despite this uncertainty, models of AN have characterised a range of social–emotional difficulties which may constitute a possible maintenance mechanism in AN, as well as a barrier to treatment (Oldershaw, Startup, & Lavender, 2019; Treasure, Corfield, & Cardi, 2012; Treasure & Schmidt, 2013). Participants with AN, when compared with healthy controls (HC), exhibit difficulties in interpreting and regulating emotion states as well as demonstrating attentional biases towards negative stimuli such as negatively valenced faces (Dapelo, Surguladze, Morris, & Tchanturia, 2015; Harrison, Sullivan, Tchanturia, & Treasure, 2010; Westwood, Kerr‐Gaffney, Stahl, & Tchanturia, 2017). Social–emotional difficulties are known to mediate the relationship between eating disorder symptoms and co‐morbid traits (Mansour et al., 2016) and it has been argued that the interaction amongst social–emotional, cognitive and interpersonal difficulties in AN contribute to the development of illness (Treasure & Schmidt, 2013), as well as being a potential risk factor for AN (Treasure et al., 2012). Recent evidence has highlighted that treatment models need to address social–emotional difficulties in order to successfully treat AN and that findings from neuroscience can help inform these models (Oldershaw et al., 2019). Therefore, fully characterising social–emotional difficulties on a neural level would contribute to a greater understanding of the biological mechanisms possibly causing and maintaining the disorder and inform treatment options.

Examining the neural underpinnings of social–emotional processing difficulties can be done using an Implicit Emotional Processing Task (IEPT) while undergoing Functional Magnetic Resonance Imaging (FMRI, Phillipou et al., 2015). Implicit rather than explicit tasks are commonly used to examine social–emotional processing while undergoing FMRI as implicit tasks are less cognitively demanding and do not interfere with emotional processing (Phillipou et al., 2015). IEPT involve showing a series of emotional faces to participants while explicitly asking participants to attend to another aspect of the face such as gender, rendering emotional processing implicit. One of the first studies to examine social emotional processing using an IEPT in AN found increased blood oxygen level dependent (BOLD) response in the fusiform gyrus in underweight individuals with AN using exploratory whole brain analysis (Fonville, Giampietro, Surguladze, Williams, & Tchanturia, 2014). The authors hypothesised that this reflected alterations in the detection/perceptual nodes of social‐processing networks, which is involved with early perceptual processing of social–emotional stimuli (Fonville et al., 2014). This hypothesis is based on the theory put forward by Nelson and colleagues who argued that social information processing can be divided into three broad functional nodes, each with a different role in social emotional processing; the perceptual, affective and cognitive regulatory/executive nodes (Nelson, Jarcho, & Guyer, 2016). The perceptual node consists of the occipital face area (OFA) and fusiform face area (Nelson et al., 2016) and has been associated with an atypical response in other psychiatric conditions such as depression (Le, Borghi, Kujawa, Klein, & Leung, 2017) and Autism Spectrum Disorder (Nomi & Uddin, 2015), both of which are over represented in individuals with AN (Haynos & Fruzzetti, 2011; Westwood & Tchanturia, 2017).

In spite of the findings from Fonville and colleagues suggesting alterations during early perceptual processing of social emotional stimuli in AN, issues still remain. Other IEPT studies of underweight individuals with AN have not replicated this result (Leppanen et al., 2017a; Leppanen et al., 2017b; Phillipou et al., 2015). There are multiple reasons why this maybe the case. Previous studies, for example, have used different experimental and analysis methods to the Fonville et al study, such as using participants own faces (Phillipou et al., 2015) rather than standardised faces, or different contrast methods (Leppanen et al., 2017a; Leppanen et al., 2017b). Previous work may have also been underpowered, which is known to cause false positive and false negative findings (Vadillo, Konstantinidis, & Shanks, 2016). Further studies, using a standardised methodology with larger sample sizes and greater statistical power are therefore needed to address inconsistencies in previous findings.

Fonville and colleagues also limited their study to the use of underweight AN participants and examined the neural response to only happy emotions. Behavioural evidence and evidence from FMRI have demonstrated that individuals with AN process negative stimuli atypically (Dapelo et al., 2015; Harrison et al., 2010; Leppanen et al., 2017a; Leppanen et al., 2017b). Therefore it is of interest to understand whether alterations in early social processing occur with negative stimuli as well as positive. Behavioural studies have also demonstrated that underweight individuals with AN and those who are weight restored have different emotional processing (Oldershaw et al., 2019). Weight‐restored individuals with AN have a differing neural profile compared to HC and those with acute illness. For example, during a social identity game, weight‐restored individuals with AN in response to directed self‐evaluations activated the dorsal anterior cingulate cortex and inferior frontal gyri response compared to underweight individuals with AN and HC (McAdams et al., 2016). The authors argued that neural differences in this social–emotional task in weight‐restored individuals may constitute a target for potential treatment (McAdams et al., 2016). This finding demonstrates the impact of malnutrition on social–emotional processing in AN. Therefore, it would be of interest to examine the effect of weight restoration on neural processing in an IEPT.

Finally, no functional connectivity paradigms using an IEPT have been conducted in individuals with AN as far as we are aware. Recent evidence has suggested that emotion is not localised to specific brain regions, but rather distributed across networks (Diano et al., 2017; Kragel & LaBar, 2016; Lindquist, Wager, Kober, Bliss‐Moreau, & Barrett, 2012; Pessoa, 2014) demonstrating functional connectivity to be important in the study of social emotional processing. Evidence from IEPTs in underweight individuals with AN has shown that alterations in the perceptual node (Fonville et al., 2014) and in the affective node (Leppanen et al., 2017a; Leppanen et al., 2017b) exist. Therefore, it would be of great interest to see if functional connectivity from these nodes to other brain regions differs between participants with lived experience of AN and healthy controls (HC) in an IEPT. An important region in the perceptual node is the OFA, which is involved in face processing (Nelson et al., 2016; Pitcher, Walsh, & Duchaine, 2011) and is located within the inferior occipital gyri (Pitcher et al., 2011). Previous face processing connectivity paradigms in AN and other disorders such as Tourette's syndrome have examined connectivity using the OFA (Moody et al., 2015; Rae et al., 2018). OFA/inferior occipital gyrus connectivity has also been shown to be modulated by emotional faces (Xiu, Geiger, & Klaver, 2015). An important region of the affective node is the amygdala, whose role in emotional processing has been well characterised (Diano et al., 2017). Previous connectivity work using the amygdala in face processing in other disorders has highlighted its importance (Goulden et al., 2012). As the OFA and amygdala are key regions of the perceptual and affective nodes respectively, these are ideal candidates to be used as seed regions in a functional connectivity paradigm, to examine how the perceptual and affective nodes interact with the rest of the brain when viewing emotional faces.

Therefore, this study will examine the neural underpinnings of emotional difficulties in individuals with AN using an IEPT. We used a similar analysis method to Fonville and colleagues in an attempt to validate previous findings as well as expanding on them by using different emotional stimuli, a larger sample size and comparing a weight‐restored (WR) group and an acutely underweight group (AAN). We have also expanded upon Fonville and colleagues' study by exploring how the perceptual and affective nodes interact with the rest of the brain by using the commonly used functional connectivity paradigm psycho‐physical interactions (PPI), which examines task‐specific increases in connectivity between a predefined seed region and other brain regions (O'Reilly, Woolrich, Behrens, Smith, & Johansen‐Berg, 2012). The seed regions for the PPI analysis were the bilateral OFA and amygdala, due to their key roles within the perceptual and affective nodes respectively. Our hypothesis was that participants with AN would have an increased BOLD response in the perceptual node, compared to HC as well as having an atypical functional connectivity profile from the perceptual and affective nodes (as represented by the OFA and amygdala) to the rest of the brain.

## 2 MATERIALS AND METHODS

### 2.1 Participants

In total 191 participants were recruited, which included 58 AAN, 60 WR and 73 HC participants. Participants' height and weight were taken before the MRI scan and were used to calculate BMI (kg/cm^2^) to allow for group stratification. For participants under 18 years old an age‐adjusted BMI formula was used, which is a percentage of median BMI for age and gender. A cut‐off of 85% of percentage of median BMI was used for group stratification based on clinical guidelines (Royal College of Psychiatrists, 2012). AAN was defined as having a concurrent diagnosis of AN as defined by DSM‐5 criteria, with a percentage of median BMI of less than 85% for participants aged under 18 or a BMI of less than 18.5 for participants aged over 18. WR was defined as having previously been diagnosed with AN, but having had a BMI within the healthy weight range (18.5–25) if over 18 years old or a percentage BMI of greater than 85% if aged under 18 years old at the time of enrolment and MRI scan. HCs were defined as having no history of an eating disorder, with a BMI within the healthy range for age at the time of the study. This resulted in three HC being excluded due to being underweight for age, leaving 70 HC. The mean age of the AAN group was 19.40 (2.83), WR 18.37 (3.59) and HC 19.37 (3.36) and did not differ between groups (see Table 1). Groups also did not differ on Intelligence Quotient (IQ, see Table 1). Patients with AN were recruited from the South London and Maudsley Specialist Eating Disorders Service, South West London and St George's Eating Disorders Service, as well as Beat, the largest UK charity for eating disorders. HC participants were recruited from the local community, as well as King's College London staff and students. Participants were eligible if they were right‐handed, aged 12 to 27, had no history of serious brain trauma, learning disabilities or neurological impairment, and no MRI incompatibility (pregnancy, claustrophobia and metal in or around the body, unable to be removed). All participants were screened using the structured clinical interview for DSM 5 researcher version for any major psychiatric co‐morbidities (First, Williams, Karg, & Spitzer, 2015).

**TABLE 1:** Participant demographics and clinical information with Kruskal Wallis tests and multiple comparisons corrected

	AAN mean (*SD*)	WR mean (*SD*)	HC mean (*SD*)	Kruskal Wallis test	Bonferroni corrected *p* values, using Mann–Whitney *U* AAN/WR AAN/HC HC/WR		
Age (in years)	19.40 (2.83)	18.37 (3.59)	19.37 (3.36)	5.58 (Kruskal test statistic) 0.061 (*p* value) 180 (df)	—	—	—
BMI	16.38 (1.39)	20.36 (2.33)	22.83 (3.36)	115.00 (Kruskal test statistic) ***<0*.*01*** (*p* value) 167 (df)	***<*.*01***	***<***.***01***	***<*** .***01***
EDE‐Q global score	3.35 (1.54)	2.88 (1.62)	0.61 (0.83)	81.54 (Kruskal test statistic) ***<*.*01*** (*p* value) 166 (df)	.13	***<*** .***01***	***<*** .***01***
HADS score	19.31 (7.78)	15.79 (7.22)	8.04 (5.59)	61.41 (Kruskal test statistic) ***<*.*01*** (*p* value) 166 (df)	.***01***	***<*** .***01***	***<*** .***01***
IQ	109.13 (6.88)	110.69 (7.57)	112.67 (7.64)	4.34 (Kruskal test statistic) .11 (*p* value) 165 (df)	—	—	—
Duration of illness (in years)	3.69 (2.81)	3.84 (2.80)	n/a		—	—	—

Abbreviations: AAN, Acute anorexia nervosa; BMI, body mass index; df, Degrees of freedom; EDE‐Q, Eating Disorder Examination–Questionnaire version; HADS, Hospital Anxiety Depression Scale; HC, Healthy control; WR, Weight‐recovered anorexia nervosa.

All participants gave written, informed, consent and the study was approved by National Research Ethics Committee (17/LO/2071). All research activities were conducted in accordance with the Declaration of Helsinki (2013).

### 2.2 Task

The implicit emotion task involved showing participants a series of faces with differing emotional qualities. Participants indicated on a button box whether the faces were male or female, to occupy conscious, explicit components of attentional processing and rendering emotional reactivity implicit. Faces were taken from a standardised series of images (Young, Perrett, Calder, Sprengelmeyer, & Ekman, 2002). The emotional facial expressions examined were neutral, happy and fearful as used in previous IEPT in AN (Fonville et al., 2014; Leppanen et al., 2017a; Leppanen et al., 2017b), allowing comparisons to be made to the previous literature. The emotional content of the faces was happy (showing a 100% happy facial expression), partially happy (showing a 50% happy, 50% neutral facial expression), neutral (a 100% neutral facial expression), partially fearful (a 50% fear, 50% neutral facial expression) and fearful (a 100% fearful facial expression). Testing was divided into tasks presented within the same MRI experimental testing session. The order of tasks was standardised, so that participants completed the testing task of partially and fully happy faces prior to completing the partially or fully fearful faces testing tasks. Twenty fully‐emotional faces (100% happy or fearful), 20 partially‐emotional faces (50% happy or fearful), 20 neutral faces and 13 fixation crosses were shown in each testing task. Faces were presented for 2 s, with a variable inter‐stimulus duration of one to 7 s, which was fixed to a Poisson distribution. Similar designs have been used in other experiments in eating disorders (Fonville et al., 2014) and mood disorders (Surguladze et al., 2010).

### 2.3 Clinical and self‐reported measures

Before the MRI scan, participants completed the self‐reported Eating Disorders Examination ‐Questionnaire (EDE‐Q, Fairburn & Beglin, 2008) and the Hospital Anxiety and Depression Scale (HADS, Zigmond & Snaith, 1983) to examine eating disorder psychopathology as well as anxiety and depression symptomatology. Participants also completed the National Adult Reading Test to measure IQ (Nelson & Wilson, 1991) and a demographic questionnaire which included duration of illness.

### 2.4 Scanning parameters

Images were acquired using a 3T GE MRI scanner, located at the Centre for Neuroimaging Sciences at King's College London. A high resolution T1 weighted image was taken for FMRI co‐registration, with the following parameters: echo time 3.016 s, repetition time 7.312 s, 1.2 mm slice thickness, field of view (FOV) 270 mm, flip angle 11°, matrix 256 × 256 pixels. T2* weighted gradient echo planar images, depicting BOLD responses, were acquired with 41 slices of 3 mm thickness and the following parameters: Repetition time of 2 s, echo time 30 ms, FOV 240 mm^2^, flip angle 75°, matrix of 64 × 64 pixels and a slice gap of 3.3 mm.

### 2.5 Statistical analysis

#### 2.5.1 Clinical and behavioural analysis

Group differences in age BMI, EDE‐Q global score, HADS and IQ were initially examined using a one‐way ANOVA; however, as the assumption of normality of residuals was violated, Kruskal Wallis tests were used for the main analysis. When significant group differences were demonstrated, post‐hoc Mann–Whitney *U* tests, Bonferroni‐corrected for multiple comparisons, were performed to test for direction of significance. A Mann–Whitney *U* test was also conducted due to violation of the normal distribution of data, to examine differences in illness duration between the AAN and the WR groups. Group differences in reaction time and number of correct responses for the task were also examined using a Kruskal Wallis test due to violation of the assumption of normally distributed residuals. All clinical and behavioural data was analysed using Python 3.8.5, in the Anaconda software packages (Anaconda software distribution, 2016).

#### 2.5.2 Neuroimaging data analysis

All FMRI data was analysed using the Statistical Parametric Mapping software 12 toolbox (SPM12, http://www.fil.ion.ucl.ac.uk/spm). The images were slice‐time corrected, motion corrected, co‐registered to a high resolution T1 image and normalised to the standard Montreal Neurological Institute (MNI) Template space. Images were spatially smoothed, with an 8 mm full width at half maximum Gaussian kernel and assessed for quality control. Two participants' scans had to be excluded due to image acquisition errors, leaving 57 AAN, 60 WR and 69 HC (186 participants) in the final analysis.

**FIGURE 1:**
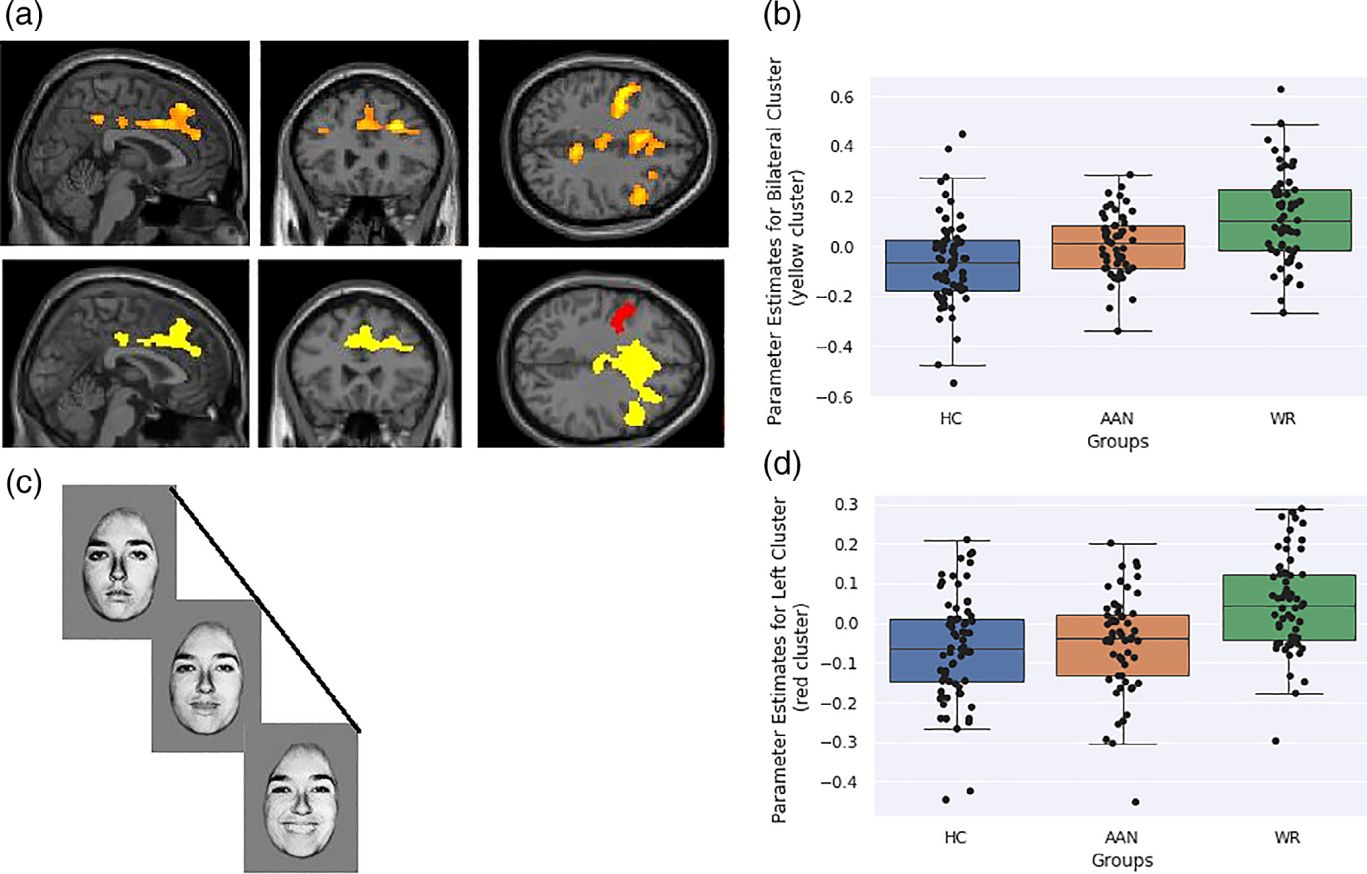
(a) Significant Blood Oxygen Level Dependent response from Psycho‐Physical Interactions analysis in a one‐way ANOVA, between all three groups, for the Happy Linear Contrast. Cluster colours are presented below for easy of interpretation. (b) Contrast estimated plotted for each group for the bilateral cluster (yellow cluster) located at 28, 32, 32. Blue is healthy controls, red is acutely underweight AN group and green is weight‐restored AN group. (c) Contrast estimates plotted for each group for left‐sided cluster (red cluster) located at −44, 0, 40 Blue is healthy controls, red is acutely underweight AN group and green is weight‐restored AN group. (d) Pictorial representation of task

Fixed‐effects analysis was carried out for each participant, using the general linear model (GLM). Regressors were used to model the onset and duration of the fixation cross, neutral faces, partially happy faces, partially fearful faces, fully happy and fully fearful faces with six regressors to control for head motion. The time series was then convolved with the canonical haemodynamic response function and a high‐pass filter of 120 s was fitted to the data. To investigate responses to emotional faces, a linear contrast was fitted to the data, consisting of neutral faces—partial emotion—full emotion. This created two contrasts; a happy linear contrast consisting of neutral faces (taken from the happy testing session)—partially happy faces—full happy faces as well as a fear linear contrast consisting of neutral faces—partially fearful faces—fully fearful faces (see Figure 1).

The PPI analysis aimed to identify brain regions where emotional‐related connectivity to the OFA differed between groups. As this approach has been used in disorders co‐morbid with AN but not directly in individuals with AN, the PPI method used in this study was similar to that from Goulden et al. (2012). First masks of the bilateral OFA and amygdala were created using the WFUpickatlas toolbox (Maldjian, Laurienti, Kraft, & Burdette, 2003) in SPM12. The co‐ordinates for the OFA were 46, −75, −3 for the right OFA and −43, −82, −7 for the left OFA (Bona, Cattaneo, & Silvanto, 2015) while an anatomical mask of the amygdala was used. Then the time series was calculated and extracted from the OFA and amygdala for each experimental condition for each participant using the first eigenvariate from the time series of all voxels in the OFA and amygdala masks. A PPI regressor was created, using the OFA and amygdala time series and a vector encoding for task comparison, which consisted of two linear contrasts, a happy linear contrast (neutral faces—partially happy face—fully happy faces) and a fear linear contrast (neutral faces—partially fearful – fully fearful faces). The PPI regressor was entered into a fixed level GLM for each participant creating PPI contrast images for the happy linear contrast and fear linear contrast. These two contrast images were brought forward for random effects analysis to test for group differences.

Random effects analysis involved one‐way ANOVAs between all three clinical groups for each of the two whole brain linear contrasts and the two PPI linear contrasts. Significance was defined as surviving cluster extent threshold of *p* < .05 family‐wise error (FWE) corrected for multiple comparisons, with an uncorrected cluster‐forming threshold of *p* < .001. If significance was found, post‐hoc *t*‐tests in SPM were used to find direction of significance along with plotting parameter estimates.

To further explore the relationship between eating disorder, depressive symptoms as well as age to the biological underpinnings in relation to emotion, parameter estimates were extracted from significant clusters, using the MaRsBar ROI toolbox (Brett, Anton, Valabregue, & Poline, 2002) in SPM12. Extracted parameter estimates were correlated using Spearman's correlation analysis with age, BMI, EDE‐Q and HADS. Correction for multiple comparisons was done by FWE correction, with *p*(FWE) < .05 considered significant. Within‐group correlations was also conducted and the results are presented in the Supporting Information. Extracted parameter values were also used to calculate effect size, using Cohen's *d*, once direction of significance was discovered in significant clusters. All statistical analysis with parameter estimates was conducted in the Anaconda environment using python 3.8.5 (Anaconda software distribution, 2016).

## 3 RESULTS

### 3.1 Self‐report and behavioural results

Participants' clinical and demographic data are presented in Table 1. All groups differed significantly on BMI and HADS. The AAN and WR groups had significantly increased EDE‐Q scores compared with HC, but did not differ from each other. Age and IQ did not significantly differ between groups. No group differences between the AAN and the WR were demonstrated for illness duration (*Mann–Whitney U statistic = 1*,*216*, *p =* .*82*, not in table).

Task‐related behavioural data revealed no group differences in reaction time to partially happy (*Kruskal test statistic = 0*.*58*, *p(FWE) = 1*.*0*), partially fearful (*Kruskal test statistic = <0*.*01*, *p(FWE) = 1*.*0*), happy (*Kruskal test statistic = 0*.*18*, *p(FWE) = 1*.*0*), neutral faces (*Kruskal test statistic = 0*.*05*, *p(FWE) = 1*.*0*) and fearful faces (*Kruskal test statistic = 0*.*22*, *p(FWE) = 1*.*0*). There were also no group differences for accuracy for partially happy (*Kruskal test statistic = 0*.*78*, *p(FWE) =* .*95*), partially fearful (*Kruskal test statistic = 3*.*60*, *p(FWE) =* .*52*), happy (*Kruskal test statistic = 0*.*18*, *p(FWE) =* .*95*), neutral faces (*Kruskal test statistic = 1*.*96*, *p(FWE) =* .*76*) and fearful faces (*Kruskal test statistic = 6*.*23*, *p(FWE) =* .*20*).

### 3.2 Neuroimaging results

#### 3.2.1 Whole brain analysis for the happy faces linear contrast

No significant whole brain group differences were demonstrated for the happy faces linear contrast.

#### 3.2.2 Whole brain analysis for the fear faces linear contrast

No significant between group differences were demonstrated for the fear faces linear contrast.

#### 3.2.3 PPI connectivity analysis

PPI analysis demonstrated significant group differences in the happy faces linear contrast between the OFA and two clusters, one frontal cluster extending bilaterally and one left‐sided frontal cluster, demonstrated in Figure 1 and Table 2. The bilateral frontal cluster covered the bilateral anterior and middle cingulate cortices as well as the bilateral superior, middle, medial and inferior frontal gyri and the right precentral gyri. The left‐sided frontal cluster extended to the inferior and middle frontal gyri as well as the precentral gyri. Post‐hoc t‐tests demonstrated the direction of significance was WR > AAN and WR > HC for both of the clusters with a small to medium effect size for both cluster. No significant group differences were seen for the fearful linear contrast or for the amygdala.

**TABLE 2:** One‐way ANOVA to show significant BOLD connectivity differences between all three groups with the Amygdala as Seed Region

Brain region	Peak MNI co‐ordinates *X*, *Y*, *Z* mm	*k*	*Z*	*p* (*FWE*)	Mean parameter estimates (*SD*)	Direction of significance from post hoc *t*‐tests (effect size, Cohen's *d*)
*Happy linear contrast*						
Bilateral anterior and middle cingulate cortices Bilateral superior frontal gyri Bilateral middle frontal gyri Bilateral medial frontal gyri Bilateral inferior frontal gyri Right precentral gyrus	28, 32, 32	3,298	5.07	<.001	AAN 0.00 (0.12) WR 0.12 (0.18) HC −0.06 (0.18)	WR > AAN (0.42) WR > HC (0.42)
Left middle frontal gyrus Left inferior frontal gyrus Left precentral gyrus	−44, 0, 40	398	4.33	.006	AAN −0.02 (0.13) WR 0.1 (0.19) HC −0.05 (0.16)	WR > AAN (0.43) WR > HC (0.43)

*Note*: Uncorrected cluster forming threshold *p* value <.001 with correction for multi comparisons by cluster extent.

Abbreviations: AAN, acute anorexia nervosa participants; FWE, family wise error; HC, healthy controls; k, cluster extent; MNI, Montreal Neurological Institute; WR, weight‐restored anorexia nervosa participants.

#### 3.2.4 Analysis of parameter estimates

We conducted correlations between age, BMI, EDE‐Q and HADS to extracted parameter estimates from the significant PPI clusters. The EDE‐Q was very weakly correlated to both clusters, *p =* .*025*, *r = 0*.*21* for the cluster located at 28, 32, 32 and *p =* .*048*, *r = 0*.*19* for the cluster located at −44, 0, 40. We also ran within‐group correlations which we have presented in the Supporting Information. No other correlations were found.

## 4 DISCUSSION

The aim of this study was to examine the neural underpinnings of social–emotional difficulties in AN, using a similar FMRI acquisition and analysis method to Fonville and colleagues. We aimed to validate their whole brain results as well as to expand on the previous study by using a larger sample size, inclusion of a negative stimuli as well as a WR group. Our hypothesis for the whole brain analysis was that the AN groups would have increased response in areas of the perceptual node, but this was not supported by our data. We also expanded on the previous study by examining functional connectivity differences between the perceptual and affective nodes to the rest of the brain. We hypothesised that the AN groups would have an atypical functional connectivity profile from the perceptual and affective nodes, which our data partially supports. We found that the WR group had increased connectivity between two clusters and the OFA compared to both AAN and HC.

In response to the positive facial emotions the WR group demonstrated increased connectivity between the OFA and the bilateral medial prefrontal cortex, the bilateral anterior cingulate cortex, inferior and middle frontal gyri. Previous work has demonstrated that these areas have been associated with top‐down emotional regulation (Etkin, Egner, & Kalisch, 2011), social cognition and cognitive control (Brewer, Garrison, & Whitfield‐Gabrieli, 2013) as well as forming part of the cognitive control network (Breukelaar et al., 2017). These regions also form a part of the cognitive regulatory node (Nelson et al., 2016). The increased connectivity from the perceptual node to cognitive regulatory node in the WR group may represent excessive cognitive processing and emotional regulation during early perceptual processing, which does not take place in AAN and HC groups. Behavioural studies lend support to this hypothesis by suggesting increased cognitive processing exist beyond the acute phase of the AN (Fuglset, 2019; Tchanturia et al., 2012) with behavioural models proposing that cognitive and social emotional difficulties act together to maintain illness (Treasure & Schmidt, 2013). Also in disorders which are co‐morbid with AN such as depression (Haynos & Fruzzetti, 2011), a similar hypothesis of atypical cognitive control response of early perception has been proposed as a key deficit (Le et al., 2017).

Interestingly we demonstrated no whole brain or connectivity differences involving the affective node. Though previous studies using IEPT in underweight AN individuals have found atypical BOLD responses in areas of the affective node, the amygdala and the insula (Leppanen et al., 2017a; Leppanen et al., 2017b), we have not been able to reproduce this. Our results therefore suggest that individuals with AN do not engage atypical affective processing of emotional stimuli. This may indicate that increased cognitive control of early perception, rather than atypical affective processing could be a key compensatory mechanism when processing happy emotional stimuli in our WR group, though further research is needed to confirm this.

Previous work in IEPTs in AN by Fonville and colleagues suggests that early perceptual differences in underweight AN individuals underlay social–emotional processing difficulties present in AN (Fonville et al., 2014). We have not replicated this study's whole brain analysis between underweight AN and HCs, as our whole brain analysis did not yield any results, while our connectivity findings suggested a difference between WR > AAN and HC. Our results do however build on Fonville and colleagues' interpretation, suggesting the neural basis of social emotional difficulties may be increased connectivity between the perceptual and cognitive regulatory nodes, possibly representing increased cognitive control of early perceptual processing of social stimuli in WR individuals and not excessive affective processing.

Interestingly eating disorder parameters were positively correlated to both the PPI clusters, so that increased eating disorder symptomatology and increased connectivity are correlated. Behavioural models of AN suggest that increased eating disorder symptoms is associated with different social–emotional experiences (Oldershaw et al., 2019) and that eating disorder symptomatology, increased cognitive control and social emotional difficulties are inter‐related (Treasure & Schmidt, 2013). Therefore the correlation between eating disorder symptoms and connectivity between the perceptual node and the cognitive regulatory node maybe the neural basis of social emotional‐difficulties in individuals with AN. Though, clearly this is a tentative hypothesis and would need to be further explored before any firm conclusions could be made.

Our results however raise some interesting questions such as why no connectivity differences were seen between the AAN and HC groups, but connectivity differences between the AN groups and between the WR and HC existed. A possible explanation could be the modulating effect of weight on brain function in emotional processing tasks. Low weight has been shown to affect different aspects of emotion, being used as an emotional regulation strategy to ease emotional regulation difficulties (Brockmeyer et al., 2012), as well as reducing emotional experience (Oldershaw et al., 2019). Theoretical models of AN propose that eating disorder behaviours which lead to low weight are used as a coping strategy for emotional arousal (Haynos & Fruzzetti, 2011). It could be that low weight affects social–emotional processing in a similar manner to emotional regulation, emotional arousal and emotional experience. Therefore, it could be that as individuals with AN gain weight, atypical functional connectivity in response to emotional stimuli emerge. This tentative hypothesis would be interesting to explore, possibly in a longitudinal manner, as individuals with AAN weight restore.

Another interesting question is why there were no between‐group differences for the exploratory whole brain analysis. Previous social–emotional experiments have demonstrated exploratory whole brain differences between underweight, weight‐restored individuals with AN and HC (McAdams et al., 2016; McAdams, Lohrenz, & Montague, 2015; Phillipou et al., 2015). A possible reasons we were unable to replicate whole brain findings from these studies could include other studies having altered the IEPT (Phillipou et al., 2015), or used different social–emotional tasks (McAdams et al., 2015; McAdams et al., 2016). Other studies may not have had the same level of statistical power as the current study, which can also cause false positives and negatives (Vadillo et al., 2016).

Our results are consistent with previous evidence demonstrating no whole brain group differences between AN and HC during social–emotional tasks (Bang, Rø, & Endestad, 2016; Cowdrey, Harmer, Park, & McCabe, 2012;Leppanen et al., 2017a; Leppanen et al., 2017b). However, like this study, when a different analytical strategy is used, group level differences emerge (Bang et al., 2016; Leppanen et al., 2017a; Leppanen et al., 2017b). It might be that current exploratory whole‐brain analysis methods used by this and other studies examining social emotional processing in individuals with AN are not sensitive enough to detect social–emotional group differences in participants with AN.

Another possible reason for our lack of whole brain group differences could be that the neural basis of social–emotional difficulties in individuals with AN may be due to network and functional connectivity disruption rather than to disruption in localised regions. This fits with recent evidence that argues emotional processing is distributed across networks (Diano et al., 2017; Kragel & LaBar, 2016; Pessoa, 2014). Therefore, as exploratory whole brain analysis cannot examine functional connectivity, this may explain why group level differences did not emerge in our study for the whole brain exploratory analysis, but did in the PPI analysis. As connectivity has not been explored in IEPT before, further research examining connectivity in social–emotional processing in AN is needed to evaluate this hypothesis.

Finally, an older age or a longer duration of illness might be needed for whole brain functional differences to emerge. Previous studies that have found group‐level whole brain differences between underweight AN and HC participants have recruited individuals who are older with a longer illness duration compared to this study (Fonville et al., 2014; McAdams et al., 2015; McAdams et al., 2016; Phillipou et al., 2015). The mean duration of illness for our AAN group was 3.69 years for the WR group 3.84 years and mean age of 19.40 year (AAN), 18.37 years (WR) and 19.37 years (HC). Compared with previous studies that used participants with AN mean age and illness duration of 27 years and 10 years (McAdams et al., 2016) and mean age and mean illness duration of 23 and 7 years (Fonville et al., 2014). Therefore the age and length of illness of our participants may be insufficient to allow for whole brain differences to emerge. It might be the case that atypical functional connectivity is present at an earlier illness stage in younger individuals with AN, which over the course of time develops into whole brain regional differences. This again would be a worthwhile area to explore with longitudinal studies.

The final notable finding is that no results for the fearful stimuli were found. This is surprising as group differences have been demonstrated in numerous negative conditions, such as fearful faces (Leppanen et al., 2017a; Leppanen et al., 2017b), in emotional conflict tasks (Bang et al., 2016) and viewing negative images (Seidel et al., 2018). However, we believe this finding to be important. Lack of response to fearful stimuli could suggest that atypical processing in AN occurs in response to positive facial emotional stimuli only. Therefore, atypical processing of positive facial stimuli could be a driving force of social–emotional difficulties, not atypical processing to fearful facial stimuli. This hypothesis would need to be further explored before any firm conclusions can be made.

This study is not without limitations. Our WR group included a relatively heterogeneous mix of participants, some who had fully recovered, however some still remained symptomatic. We also did not have knowledge of the clinical course of the AAN group. This group was recruited from a variety of services and it was unclear if some participants in this group were engaged in treatment and improving or deteriorating in nutritional status. Underweight individuals engaging with treatment, for example, will be at a different stage of the disorder compared to underweight individuals who are not. This may have introduced heterogeneity into this group and may offer an explanation for some of our findings. We would recommend that future research control for clinical course of the disorder.

The use of static faces in this study can also be considered a limitation. Dynamic facial expressions have been shown to be more sensitive at depicting subtle facial expressions of emotion (Kätsyri & Sams, 2008) and are considered more natural, which might be better in patient groups with known difficulties in extracting information from stimuli, such as in ASD (Dobs, Bülthoff, & Schultz, 2018). Future studies may wish to consider using dynamic faces, either to try to replicate IEPT findings in AN, or as an adjunct to convey subtler emotional expressions.

Another limitation of the study is that we could not control for medication or AN subtype in the analysis, as this information was not known. Medication has been demonstrated previously to affect facial processing in AN (Fonville et al., 2014), and in emotional tasks AN subtype has been shown to affect neural processing (Miyake et al., 2010). Future research may wish to take medication and AN subtype into consideration.

Future research may also consider adapting the IEPT. While faces are the most powerful tool for communicating affective stimuli (Diano et al., 2017), recent evidence has shown that body cues are also used to discriminate emotions (Aviezer, Trope, & Todorov, 2012). In the context of AN, this may be of interest as images of bodies have shown to elicit an emotional response (Friederich et al., 2010), with differences in the insula (Friederich et al., 2010) and the amygdala (Vocks et al., 2010) being demonstrated. These regions have also been associated with implicit emotional processing of faces in AN (Leppanen et al., 2017a; Leppanen et al., 2017b). It may therefore be interesting for future studies to consider including both faces and bodies in emotional processing tasks.

Despite the limitations of this study, our results represent the largest sample to have investigated implicit emotional processing in young people with AN, which has important implications for future AN research. Our results contribute to a growing literature base establishing the pathophysiology of AN. By using a combination of whole brain analysis and a connectivity paradigm not previously used in IEPTs in AN, we have identified neural correlates of emotional processing difficulties. We have also suggested a unique connectivity and neural profile for WR and AAN participants, which warrants further exploration in future studies.

## Supporting information


**Appendix** **S1**: Supporting information.

## Data Availability

Data is available upon request from the authors.
