# Supplementary material for: The emotional face of anorexia nervosa: The neural correlates of emotional processing
Source: Hum Brain Mapp. 2021 Mar 19;42(10):3077–87. doi: 10.1002/hbm.25417 (PMC8193512; doi:10.1002/hbm.25417)
Supplement: Supplementary file 1 — Appendix S1: Supporting information. [file HBM-42-3077-s001.docx]

**Supplementary Material**

Table 1. Task-related behavioural data for each group by testing Task.

| Testing Task | Group | Reaction time  Mean in milliseconds (SD) | Accuracy on discerning gender of face  Mean percentage (SD) |
| --- | --- | --- | --- |
| Happy Faces | AAN | 961.57  (204.53) | 88.51  (3.40) |
|  | WR | 962.74  (212.32) | 88.58  (3.07) |
|  | HC | 963.62  (243.62) | 88.33  (3.60) |
| Partially Happy Faces | AAN | 962.28  (230.85) | 87.02  (4.21) |
|  | WR | 962.06  (217.54) | 86.83  (4.11) |
|  | HC | 981.80  (242.52) | 87.53  (3.39) |
| Neutral Faces | AAN | 968.97  (235.13) | 87.28  (5.27) |
|  | WR | 963.07  (242.22) | 88.17  (4.11) |
|  | HC | 968.19  (246.35) | 88.55  (3.84) |
| Partially Fear Faces | AAN | 929.26  (220.47) | 86.32  (6.72) |
|  | WR | 919.40  (209.47) | 88.50  (3.93) |
|  | HC | 917.91  (224.30) | 87.61  (4.25) |
| Fearful faces | AAN | 950.48  (219.59) | 85.09  (7.29) |
|  | WR | 960.44  (209.86) | 87.25  (6.07) |
|  | HC | 949.82  (240.29) | 87.90  (4.15) |
| Note. AAN = acute anorexia nervosa; BMI = body mass index; HC = healthy control; SD=Standard Deviation; WR = weigh-recovered anorexia nervosa | | | |

Table 2. Spearman’s Correlation Within-Group between Extracted Parameter Estimates for the Happy Linear Contrast between the Occipital Face Area and cluster one (28, 32, 32) and Clinical Symptoms.

| HC | | |
| --- | --- | --- |
| Independent variable | Rho | P(FWE) value |
| BMI | 0.16 | 0.92 |
| EDE-Q global score | -0.06 | 1.0 |
| HADS | <0.12 | 0.76 |
| Age | -0.13 | 0.96 |
| AAN | | |
| BMI | -0.13 | 0.97 |
| Illness Duration | -0.06 | 1.0 |
| EDE-Q global score | 0.23 | 0.75 |
| HADS | 0.20 | 0.84 |
| Age | <0.01 | 1.0 |
| WR | | |
| BMI | 0.20 | 0.8 |
| Illness Duration | 0.11 | 0.98 |
| EDE-Q global score | <-0.01 | 1.0 |
| HADS | 0.04 | 1.0 |
| Age | 0.05 | 1.0 |
| Note. AAN = acute anorexia nervosa; BMI = body mass index; EDE-Q = eating disorder examination–questionnaire version; HADS= hospital anxiety depression scale; HC = healthy control; WR = weight-recovered anorexia nervosa | | |

Table 3. Spearman’s Correlation Within-Group between Extracted Parameter Estimates for the Happy Linear Contrast between the Occipital Face Area and cluster two (-44 0 40) and Clinical Symptoms.

| HC | | |
| --- | --- | --- |
| Independent variable | Rho | P(FWE) value |
| BMI | 0.27 | 0.36 |
| EDE-Q global score | -0.06 | 1.0 |
| HADS | -0.13 | 0.97 |
| Age | 0.02 | 1.0 |
| AAN | | |
| BMI | -0.08 | 1.0 |
| Illness Duration | -0.11 | 1.0 |
| **EDE-Q global score** | **0.40** | **0.04** |
| HADS | 0.15 | 0.96 |
| Age | 0.06 | 1.0 |
| WR | | |
| BMI | 0.02 | 1.0 |
| Illness Duration | -0.17 | 0.96 |
| EDE-Q global score | -0.03 | 1.0 |
| HADS | 0.08 | 1.0 |
| Age | -0.05 | 1.0 |
| Note. AAN = acute anorexia nervosa; BMI = body mass index; EDE-Q = eating disorder examination–questionnaire version; HADS= hospital anxiety depression scale; HC = healthy control; WR = weight-recovered anorexia nervosa | | |

Table 4. Spearman’s Correlation For Extracted Parameter Estimates From the Happy Linear Contrast Between the Occipital Face Area and Cluster one (28, 32, 32) and Clinical Symptoms.

| Variable | Rho | P(FWE) |
| --- | --- | --- |
| BMI | 0.00 | 0.909 |
| **EDE-Q** | **0.21** | **0.025** |
| HADS | 0.14 | 0.166 |
| Age | -0.12 | 0.175 |
| Note. AAN = acute anorexia nervosa; BMI = body mass index; EDE-Q = eating disorder examination–questionnaire version; HADS= hospital anxiety depression scale; HC = healthy control; WR = weight-recovered anorexia nervosa | | |

Table 5. Spearman’s Correlation For Extracted Parameter Estimates From the Happy Linear Contrast between the Occipital Face Area and Cluster two (-44 0 40) and Clinical Symptoms.

| Variable | Rho | P(FWE) |
| --- | --- | --- |
| BMI | 0.04 | 0.563 |
| **EDE-Q** | **0.19** | **0.048** |
| HADS | 0.12 | 0.340 |
| Age | -0.07 | 0.556 |
| Note. AAN = acute anorexia nervosa; BMI = body mass index; EDE-Q = eating disorder examination–questionnaire version; HADS= hospital anxiety depression scale; HC = healthy control; WR = weight-recovered anorexia nervosa | | |


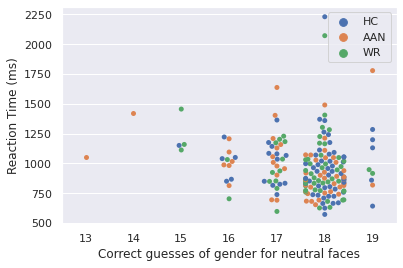
Figure S1: Plot for accuracy of neutral faces and reaction time for all three groups.


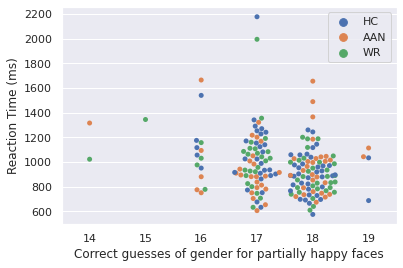
Figure S2: Plot for accuracy of happy faces and reaction time for all three groups.


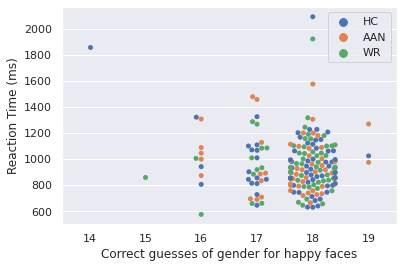
Figure S3: Plot for accuracy of partially happy faces and reaction time for all three groups.


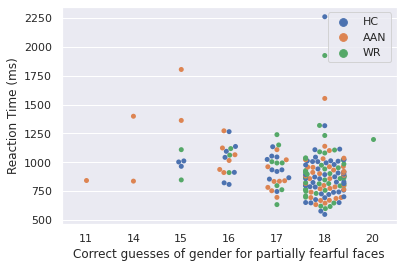
Figure S4: Plot for accuracy of partially fearful faces and reaction time for all three groups.


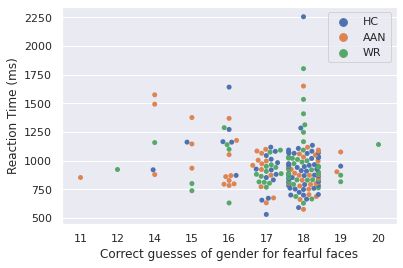
Figure S5: Plot for accuracy of fearful faces and reaction time for all three groups.
